# Phase 3 CLEAR study in patients with advanced renal cell carcinoma: outcomes in subgroups for the lenvatinib-plus-pembrolizumab and sunitinib arms

**DOI:** 10.3389/fonc.2023.1223282

**Published:** 2023-08-16

**Authors:** Viktor Grünwald, Thomas Powles, Masatoshi Eto, Evgeny Kopyltsov, Sun Young Rha, Camillo Porta, Robert Motzer, Thomas E. Hutson, María José Méndez-Vidal, Sung-Hoo Hong, Eric Winquist, Jeffrey C. Goh, Pablo Maroto, Tomas Buchler, Toshio Takagi, Joseph E. Burgents, Rodolfo Perini, Cixin He, Chinyere E. Okpara, Jodi McKenzie, Toni K. Choueiri

**Affiliations:** ^1^ Clinic for Medical Oncology and Clinic for Urology, University Hospital Essen, Essen, Germany; ^2^ Barts Cancer Institute and the Royal Free Hospital, Queen Mary University of London, London, United Kingdom; ^3^ Department of Urology, Kyushu University, Fukuoka, Japan; ^4^ State Institution of Healthcare Regional Clinical Oncology Dispensary, Omsk, Russia; ^5^ Department of Internal Medicine, Yonsei Cancer Center, Yonsei University Health System, Seoul, Republic of Korea; ^6^ Department of Biomedical Sciences and Human Oncology, University of Bari ‘A. Moro’, Bari, Italy; ^7^ Department of Medicine, Memorial Sloan Kettering Cancer Center, New York, NY, United States; ^8^ Medical Oncology, Texas Oncology, Dallas, TX, United States; ^9^ Department of Oncology, Maimonides Institute for Biomedical Research of Córdoba (IMIBIC) Hospital Universitario Reina Sofía, Córdoba, Spain; ^10^ Department of Urology, Seoul St. Mary’s Hospital, The Catholic University of Korea, Seoul, Republic of Korea; ^11^ Department of Oncology, University of Western Ontario, London, ON, Canada; ^12^ ICON Research, South Brisbane & University of Queensland, St Lucia, QLD, Australia; ^13^ Department of Medical Oncology, Hospital de la Santa Creu i Sant Pau, Barcelona, Spain; ^14^ Department of Oncology, Charles University and Thomayer University Hospital, Prague, Czechia; ^15^ Department of Urology, Tokyo Women’s Medical University, Tokyo, Japan; ^16^ Global Clinical Development, Merck & Co., Inc., Rahway, NJ, United States; ^17^ Clinical Research, Merck & Co., Inc., Rahway, NJ, United States; ^18^ Biostatistics, Eisai Inc., Nutley, NJ, United States; ^19^ Clinical Research, Eisai Ltd., Hatfield, United Kingdom; ^20^ Clinical Research, Eisai Inc., Nutley, NJ, United States; ^21^ Department of Medical Oncology, Dana-Farber Cancer Institute, Boston, MA, United States

**Keywords:** renal cell carcinoma, lenvatinib, pembrolizumab, sunitinib, bone metastases, liver metastases, lung metastases, sarcomatoid histology

## Abstract

**Introduction:**

The phase 3 CLEAR study demonstrated that lenvatinib plus pembrolizumab significantly improved efficacy versus sunitinib as first-line treatment for patients with advanced renal cell carcinoma (RCC). Prognostic features including presence and/or site of baseline metastases, prior nephrectomy, and sarcomatoid features have been associated with disease and treatment success. This subsequent analysis explores outcomes in patients with or without specific prognostic features.

**Methods:**

In CLEAR, patients with clear cell RCC were randomly assigned (1:1:1) to receive either lenvatinib (20 mg/day) plus pembrolizumab (200 mg every 3 weeks), lenvatinib (18 mg/day) plus everolimus (5 mg/day), or sunitinib alone (50 mg/day, 4 weeks on, 2 weeks off). In this report, progression-free survival (PFS), overall survival (OS), and objective response rate (ORR) were all assessed in the lenvatinib-plus-pembrolizumab and the sunitinib arms, based on baseline features: lung metastases, bone metastases, liver metastases, prior nephrectomy, and sarcomatoid histology.

**Results:**

In all the assessed subgroups, median PFS was longer with lenvatinib-plus-pembrolizumab than with sunitinib treatment, notably among patients with baseline bone metastases (HR 0.33, 95% CI 0.21–0.52) and patients with sarcomatoid features (HR 0.39, 95% CI 0.18–0.84). Median OS favored lenvatinib plus pembrolizumab over sunitinib irrespective of metastatic lesions at baseline, prior nephrectomy, and sarcomatoid features. Of interest, among patients with baseline bone metastases the HR for survival was 0.50 (95% CI 0.30–0.83) and among patients with sarcomatoid features the HR for survival was 0.91 (95% CI 0.32–2.58); though for many groups, median OS was not reached. ORR also favored lenvatinib plus pembrolizumab over sunitinib across all subgroups; similarly, complete responses also followed this pattern.

**Conclusion:**

Efficacy outcomes improved following treatment with lenvatinib-plus-pembrolizumab versus sunitinib in patients with RCC—irrespective of the presence or absence of baseline lung metastases, baseline bone metastases, baseline liver metastases, prior nephrectomy, or sarcomatoid features. These findings corroborate those of the primary CLEAR study analysis in the overall population and support lenvatinib plus pembrolizumab as a standard of care in 1L treatment for patients with advanced RCC.

**Clinical trial registration:**

ClinicalTrials.gov, identifier NCT02811861

## 1 Introduction

Kidney cancer is one of the most frequently diagnosed cancers in the United States, and the activity of traditional cytotoxic chemotherapy is limited in patients with metastatic renal cell carcinoma (RCC) (1). The phase 3 multicenter, open-label, randomized, CLEAR study (Study 307/KEYNOTE-581) compared the efficacy and safety of lenvatinib plus pembrolizumab or everolimus versus sunitinib as a first-line treatment for patients with advanced RCC (2). In the primary analysis of CLEAR, lenvatinib plus pembrolizumab demonstrated statistically significant and clinically meaningful improvement in efficacy outcomes versus sunitinib (2): median progression-free survival (PFS) was 24 months versus 9 months, respectively (hazard ratio [HR] 0.39; 95% confidence interval [CI] 0.32–0.49; *P* < 0.001); median overall survival (OS) was not reached for either arm (HR 0.66; 95% CI 0.49–0.88; *P* = 0.005); and objective response rate (ORR) was 71% versus 36%, respectively (relative risk with lenvatinib plus pembrolizumab versus sunitinib, 1.97; 95% CI 1.69–2.29) (2). The median duration of survival follow-up in the CLEAR study was 26.6 months. The safety profile for the lenvatinib plus pembrolizumab combination was consistent with that of the monotherapies, with no new safety signals.

While CLEAR showed statistically significant and clinically meaningful efficacy in the overall population, some clinical characteristics of patients with RCC can impact disease status and may play a role in physicians’ treatment decisions and patient outcomes. Such characteristics include the presence/location of metastases (specifically lung, bone, and liver), whether or not patients had a nephrectomy prior to treatment, and the presence or absence of sarcomatoid features, and these may all be considered prognostic indicators of the disease (3–5). The most common sites of metastasis in patients with RCC are lung, bone, lymph nodes, and liver (3, 6, 7). The lung is the most common site of metastasis in patients with clear cell RCC (3, 8); these patients often have more promising OS durations (median 25.1 months, 95% CI 24.1–26.0) than patients who have RCC with metastases to other sites (3, 8). Bone metastases are associated with skeletal-related events (ie, fractures, spinal cord compression, hypercalcemia, and bone pain) that impair quality of life and can lead to lower rates of survival (9–11). In a characterization of metastatic sites in patients with RCC that accessed data from more than 11000 patients from the International mRCC Database Consortium, median OS among patients with bone metastases was 19.4 months (95% CI 18.1–20.5) (3). While liver metastases are less common than bone or lung metastases, prognoses and OS rates are poor among patients with liver metastases (median OS 17.6 months; 95% CI 16.0–19.2) (3, 12).

Although localized RCC is initially treated by nephrectomy, with adjuvant pembrolizumab treatment depending on disease state and/or histology (7), 25% to 30% of patients develop metastatic disease following nephrectomy (13, 14); until recently, cytoreductive nephrectomy was widely used in the case of metastatic disease (15). While partial nephrectomy can be preferred to radical nephrectomy, it is generally considered not suitable for patients with advanced tumors (7). Sarcomatoid features can occur in most histologic subtypes of RCC (5, 16). Patients who have RCC with a sarcomatoid component (in approximately 20% of tumors from patients with advanced RCC) have a poor prognosis and low 5-year survival rate compared with patients without sarcomatoid features (5, 12, 16, 17), and treatment options for these patients is an important unmet need (18).

This analysis explored efficacy outcomes in subgroups of patients with or without specific baseline features (ie, lung metastases, bone metastases, liver metastases, prior nephrectomy, and sarcomatoid histology) using data from the lenvatinib plus pembrolizumab and sunitinib arms of CLEAR.

## 2 Methods

### 2.1 Patients

In CLEAR, patients were randomly assigned (1:1:1) to receive either lenvatinib 20 mg orally once daily plus pembrolizumab 200 mg intravenously once every 3 weeks; lenvatinib 18 mg plus everolimus 5 mg orally once daily; or sunitinib 50 mg orally once daily (4 weeks on/2 weeks off). Key eligibility criteria included: advanced RCC with no prior systemic therapy; at least 1 measurable lesion per Response Evaluation Criteria In Solid Tumors version 1.1 (RECIST v1.1); and a Karnofsky performance-status score ≥ 70. Additional eligibility criteria have been published (2). Randomization was stratified by geographic region (ie, Western Europe and North America or rest of the world) and by Memorial Sloan Kettering Cancer Center prognostic risk group (ie, favorable, intermediate, or poor risk).

### 2.2 Study design

CLEAR was a multicenter, open-label, randomized study that compared efficacy and safety of lenvatinib plus pembrolizumab or everolimus versus sunitinib in patients with RCC. The primary endpoint was PFS, as assessed by an independent review committee (IRC) using RECIST v1.1. Key secondary endpoints included OS, and ORR as assessed by IRC using RECIST v1.1. Results of the primary and key secondary endpoints have been published (2).

The trial was conducted in accordance with the International Council for Harmonisation Good Clinical Practice Guidelines and the principles of the 2013 Declaration of Helsinki. Institutional review boards or independent ethics committees approved the protocol and appropriate related documents; all patients provided written informed consent. Safety and efficacy data were monitored by an independent data and safety monitoring committee.

### 2.3 Statistics

Median PFS and OS for the lenvatinib-plus-pembrolizumab and sunitinib arms were estimated using the Kaplan–Meier method; HR and 95% CIs comparing lenvatinib-plus-pembrolizumab versus sunitinib arms were estimated by a stratified Cox model. If a stratification factor was itself a subgroup, this factor was removed from the stratified analysis. The subgroups/strata with sample size less than 5% of the treatment group are not displayed. Odds ratios were used to compare ORRs for the lenvatinib-plus-pembrolizumab and sunitinib arms. This preplanned subgroup analysis compared PFS, OS, and ORR in the lenvatinib-plus-pembrolizumab arm versus the sunitinib arm, based on selected baseline features comprising lung metastases, bone metastases, liver metastases, prior nephrectomy, and sarcomatoid histology. Patients could be included in multiple categories simultaneously. Programmed cell death ligand-1 (PD-L1) status may be another prognostic factor that can inform clinical decisions; however, data on PD-L1 status have previously been published (2) and are not included here. Data by International Metastatic Renal Cell Carcinoma Database Consortium risk group and Memorial Sloan Kettering Cancer Center risk group are included for reference. All results of this subgroup analysis are descriptive.

## 3 Results

### 3.1 Patients

**Figure 1:**
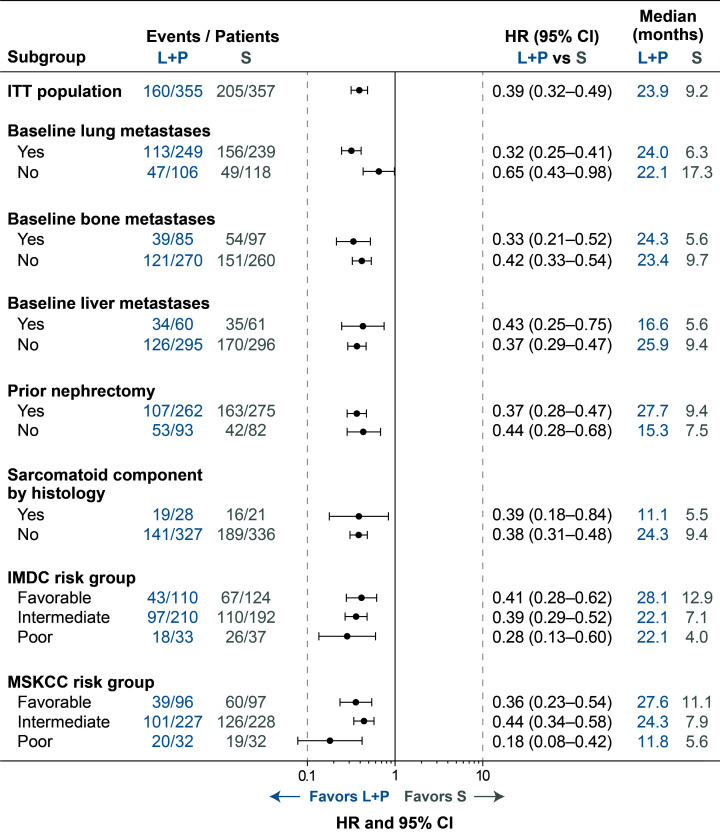
Forest Plot of PFS for Lenvatinib + Pembrolizumab Versus Sunitinib Treatment by IRC per RECIST v1.1. CI, confidence interval; HR, hazard ratio; IMDC, International Metastatic Renal Cell Carcinoma Database Consortium; IRC, independent review committee; ITT, intention to treat; L+P, lenvatinib + pembrolizumab; MSKCC, Memorial Sloan Kettering Cancer Center; PFS, progression-free survival; RECIST v1.1, Response Evaluation Criteria In Solid Tumors version 1.1; S, sunitinib.

**Figure 2:**
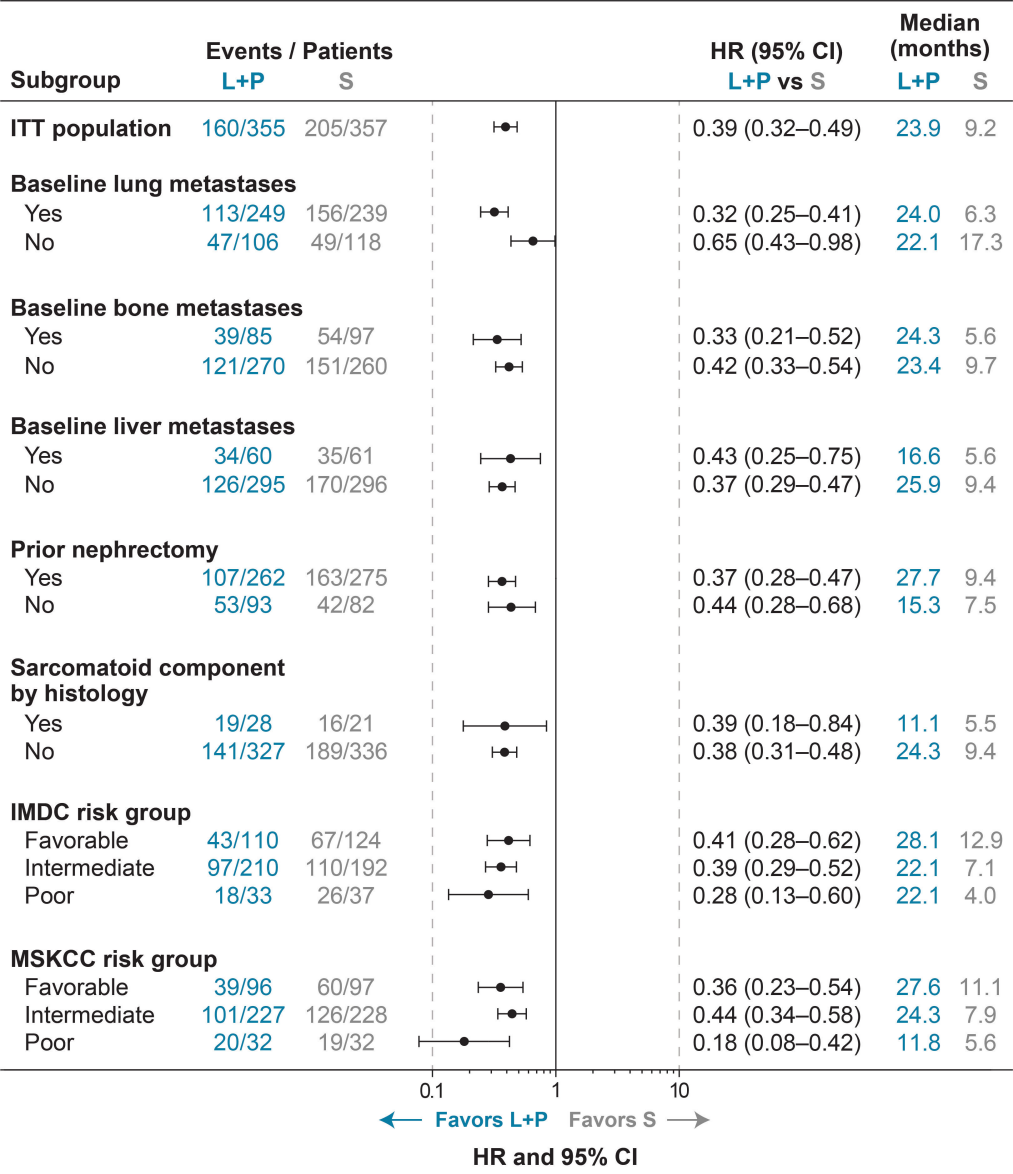
Forest Plot of OS for Lenvatinib + Pembrolizumab Versus Sunitinib Treatment. CI, confidence interval; HR, hazard ratio; IMDC, International Metastatic Renal Cell Carcinoma Database Consortium; ITT, intention to treat; L+P, lenvatinib + pembrolizumab; MSKCC, Memorial Sloan Kettering Cancer Center; NE, not estimable; OS, overall survival; S, sunitinib.

**Figure 3:**
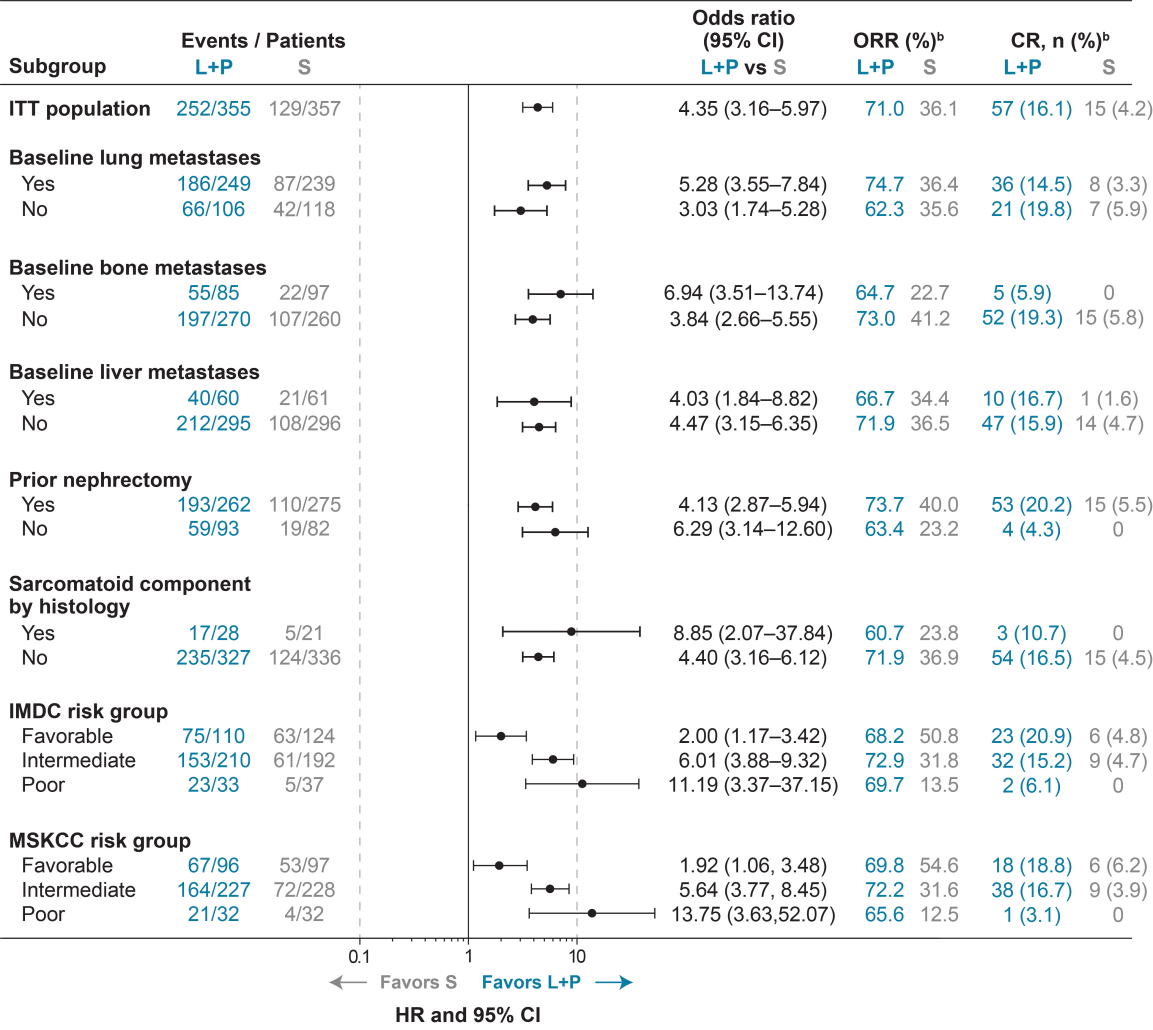
ORR^a^ and Odds Ratios for Lenvatinib + Pembrolizumab Versus Sunitinib Treatment in Subgroups of Interest. ^a^As assessed by IRC per RECIST v1.1. ^b^Percents were calculated based on listed subgroups. CI, confidence interval; CR, complete response; IMDC, International Metastatic Renal Cell Carcinoma Database Consortium; IRC, independent review committee; ITT, intention to treat; L+P, lenvatinib + pembrolizumab; MSKCC, Memorial Sloan Kettering Cancer Center; ORR, objective response rate; RECIST v1.1, Response Evaluation Criteria In Solid Tumors version 1.1; S, sunitinib.

Of the 1069 patients randomly assigned to treatment in CLEAR, 355 were randomly assigned to receive lenvatinib plus pembrolizumab and 357 to receive sunitinib (2). Baseline characteristics of patients in these 2 arms have previously been published (2), and are summarized in **Table 1**. Among patients in the lenvatinib-plus-pembrolizumab arm, 249 (70.1%) had lung metastases, 85 (23.9%) had bone metastases, and 60 (16.9%) had liver metastases. In the sunitinib arm, 239 (66.9%) patients had lung metastases, 97 (27.2%) had bone metastases, and 61 (17.1%) had liver metastases. Of the patients who had bone metastases (85 patients in the lenvatinib-plus-pembrolizumab arm and 97 patients in the sunitinib arm), 11 (12.9% of patients with bone metastasis in the lenvatinib-plus-pembrolizumab arm) and 21 (21.6% of patients with bone metastasis in the sunitinib arm), received concomitant bone-targeting treatment. Most patients (262 [73.8%] in the lenvatinib-plus-pembrolizumab arm and 275 [77.0%] in the sunitinib arm) had undergone a prior nephrectomy (2). Sarcomatoid features were observed in 28 (7.9%) patients in the lenvatinib-plus-pembrolizumab arm and in 21 (5.9%) patients in the sunitinib arm (2). The number of patients with MSKCC and International Metastatic RCC Database Consortium (IMDC) favorable/intermediate/poor risk in the lenvatinib-plus-pembrolizumab arm (MSKCC: 96 [27.0%]/227 [63.9%]/32 [9.0%]; IMDC: 110 [31.0%]/210 [59.2%]/33 [9.3%]) and sunitinib arm (MSKCC: 97 [27.2%]/228 [63.9%]/32 [9.0%]; IMDC: 124 [34.7%]/192 [53.8%]/37 [10.4%]) have been previously reported (2). Data for these groups are included for reference (**Table 1**; **Figures 1**–**3**; **Supplementary Table 1**) throughout.

**Table 1:** Patient demographics and characteristics of the lenvatinib + pembrolizumab and sunitinib arms in CLEAR^a^.

Characteristic	Lenvatinib + Pembrolizumab (n = 355)	Sunitinib (n = 357)
Median age, years (range)	64 (34, 88)	61 (29, 82)
Geographic region, n (%) Western Europe and North America Rest of world	198 (55.8) 157 (44.2)	199 (55.7) 158 (44.3)
MSKCC prognostic risk group, n (%) Favorable Intermediate Poor	96 (27.0) 227 (63.9) 32 (9.0)	97 (27.2) 228 (63.9) 32 (9.0)
IMDC risk group, n (%) Favorable Intermediate Poor	110 (31.0) 210 (59.2) 33 (9.3)	124 (34.7) 192 (53.8) 37 (10.4)
PD-L1 combined positive score, n (%) ≥1 <1 Not available	107 (30.1) 112 (31.5) 136 (38.3)	119 (33.3) 103 (28.9) 135 (37.8)
Number of metastatic organs or sites^b^, n (%) 1 ≥2	97 (27.3) 254 (71.5)	108 (30.3) 246 (68.9)
Lung metastases^b,c^, n (%)	249 (70.1)	239 (66.9)
Bone metastases^b,d^, n (%)	85 (23.9)	97 (27.2)
Liver metastases^b,e^, n (%)	60 (16.9)	61 (17.1)
Prior nephrectomy, n (%)	262 (73.8)	275 (77.0)
Sarcomatoid features, n (%)	28 (7.9)	21 (5.9)

^a^ Motzer et al., 2021 previously reported baseline characteristics in full. Table adapted from (2). ^b^As assessed by the investigators. ^c^In the lenvatinib + pembrolizumab arm, lung metastases occurred in the following IMDC risk groups: favorable: 68/110 (61.8%), intermediate: 152/210 (72.4%), poor: 27/33 (81.8%), not evaluable for risk group: 2 patients; in the sunitinib arm, lung metastases occurred in the following IMDC risk groups: favorable: 71/124 (57.3%), intermediate: 137/192 (71.4%), poor: 29/37 (78.4%), not evaluable for risk group: 2 patients. ^d^In the lenvatinib + pembrolizumab arm, bone metastases occurred in the following IMDC risk groups: favorable: 15/110 (13.6%), intermediate: 57/210 (27.1%), poor: 13/33 (39.4%); in the sunitinib arm, bone metastases occurred in the following IMDC risk groups: favorable: 24/124 (19.4%), intermediate: 56/192 (29.2%), poor: 16/37 (43.2%), not evaluable for risk group: 1 patient. ^e^In the lenvatinib + pembrolizumab arm, liver metastases occurred in the following IMDC risk groups: favorable: 15/110 (13.6%), intermediate: 34/210 (16.2%), poor: 11/33 (33.3%); in the sunitinib arm, liver metastases occurred in the following IMDC risk groups: favorable: 23/124 (18.5%), intermediate: 26/192 (13.5%), poor: 9/37 (24.3%), not evaluable for risk group: 3 patients.

IMDC, International Metastatic Renal Cell Carcinoma Database Consortium; MSKCC, Memorial Sloan Kettering Cancer Center; PD-L1, programmed cell death ligand-1.

### 3.2 Efficacy

#### 3.2.1 Progression-free survival

The data cutoff date for the primary analysis of CLEAR was August 28, 2020 (2). Median PFS, as assessed by IRC per RECIST v1.1, was longer with lenvatinib-plus-pembrolizumab versus sunitinib treatment across baseline-characteristic subgroups of interest (**Figure 1**). Among patients with baseline lung metastases, those in the lenvatinib-plus-pembrolizumab arm had a median PFS of 24.0 months and those in the sunitinib arm had a median PFS of 6.3 months (HR 0.32; 95% CI 0.25–0.41). Patients with no baseline lung metastases had median PFS of 22.1 months in the lenvatinib-plus-pembrolizumab arm and 17.3 months in the sunitinib arm (HR 0.65; 95% CI 0.43–0.98). Patients with bone metastases had a median PFS of 24.3 months versus 5.6 months in the lenvatinib-plus-pembrolizumab versus sunitinib arms, respectively (HR 0.33; 95% CI 0.21–0.52); those without bone metastases had a median PFS of 23.4 months in the lenvatinib-plus-pembrolizumab arm and 9.7 months in the sunitinib arm (HR 0.42; 95% CI 0.33–0.54). Patients with liver metastases had a median PFS of 16.6 months in the lenvatinib-plus-pembrolizumab arm and 5.6 months in the sunitinib arm (HR 0.43; 95% CI 0.25–0.75); those without liver metastases had a median PFS of 25.9 months in the lenvatinib-plus-pembrolizumab arm and 9.4 months in the sunitinib arm (HR 0.37; 95% CI 0.29–0.47).

In patients with prior nephrectomy, PFS outcomes also favored lenvatinib plus pembrolizumab (median 27.7 months) versus sunitinib (median 9.4 months) (HR 0.37; 95% CI 0.28–0.47); similarly, in those without prior nephrectomy, median PFS was 15.3 versus 7.5 months, respectively (HR 0.44; 95% CI 0.28–0.68; **Figure 1**). In patients with sarcomatoid features, median PFS was 11.1 months in the lenvatinib-plus-pembrolizumab arm versus 5.5 months in the sunitinib arm (HR 0.39; 95% CI 0.18–0.84), and in those without sarcomatoid features median PFS was 24.3 months in patients in the lenvatinib-plus-pembrolizumab arm versus 9.4 months in the sunitinib arm (HR 0.38; 95% CI 0.31–0.48).

#### 3.2.2 Overall survival

While median OS was not reached in most groups, OS results also generally favored lenvatinib-plus-pembrolizumab versus sunitinib treatment across baseline-characteristic subgroups of interest (**Figure 2**). Lenvatinib-plus-pembrolizumab treatment was favored over sunitinib treatment in patients who had baseline lung metastases (HR 0.57; 95% CI 0.40–0.80), and was favored in patients who did not have baseline lung metastases (HR 0.84; 95% CI 0.47–1.49). In patients with bone metastases, lenvatinib plus pembrolizumab was favored over sunitinib (HR 0.50; 95% CI 0.30–0.83); in patients without bone metastases, median OS was not reached in either arm (HR 0.79; 95% CI 0.54–1.14). Lenvatinib plus pembrolizumab was favored over sunitinib whether patients had liver metastases (HR 0.52; 95% CI 0.27–0.99) or not (HR 0.66; 95% CI 0.47–0.93).

Median OS favored lenvatinib-plus-pembrolizumab over sunitinib treatment both in patients with prior nephrectomy (median not reached in either group; HR 0.71; 95% CI 0.49–1.03) and without prior nephrectomy (median 33.1 versus 24.0 months, respectively; HR 0.52; 95% CI 0.31–0.86; **Figure 2**). Median OS was not reached in either treatment arm in patients with (HR 0.91; 95% CI 0.32–2.58) or without sarcomatoid features (HR 0.64; 95% CI 0.47–0.87).

#### 3.2.3 Objective response

ORR results favored lenvatinib-plus-pembrolizumab versus sunitinib treatment across subgroups of interest (ie, lung metastases, bone metastases, liver metastases, prior nephrectomy, and sarcomatoid features) (**Figure 3**, **Supplementary Table 1**). In patients with lung metastases, ORR was 74.7% in the lenvatinib-plus-pembrolizumab arm and 36.4% in the sunitinib arm (odds ratio 5.28; 95% CI 3.55–7.84). In patients without lung metastases, ORR was 62.3% in the lenvatinib-plus-pembrolizumab arm and 35.6% in the sunitinib arm (odds ratio 3.03; 95% CI 1.74–5.28). In patients with bone metastases, ORR was 64.7% in the lenvatinib-plus-pembrolizumab arm and 22.7% in the sunitinib arm (odds ratio 6.94; 95% CI 3.51–13.74). In patients without bone metastases, ORR was 73.0% in the lenvatinib-plus-pembrolizumab arm and 41.2% in the sunitinib arm (odds ratio 3.84; 95% CI 2.66–5.55). ORR was 66.7% in patients with liver metastases in the lenvatinib-plus-pembrolizumab arm and 34.4% in patients with liver metastases in the sunitinib arm (odds ratio 4.03; 95% CI 1.84–8.82). In patients without liver metastases, ORR was 71.9% and 36.5% in the lenvatinib-plus-pembrolizumab and sunitinib arms, respectively (odds ratio 4.47; 95% 3.15–6.35).

ORR was 73.7% and 40.0% in patients who had a prior nephrectomy in the lenvatinib-plus-pembrolizumab and sunitinib arms, respectively (odds ratio 4.13; 95% CI 2.87–5.94); and 63.4% and 23.2%, respectively, in patients who had not had a prior nephrectomy (odds ratio 6.29; 95% CI 3.14–12.60) (**Figure 3**, **Supplementary Table 1**). In patients with sarcomatoid features, ORR was 60.7% and 23.8% in the lenvatinib-plus-pembrolizumab arm and the sunitinib arm, respectively (odds ratio 8.85; 95% CI 2.07–37.84). In patients without sarcomatoid features, ORR was 71.9% in the lenvatinib-plus-pembrolizumab arm and 36.9% in the sunitinib arm (odds ratio 4.40; 95% CI 3.16–6.12). The rate of patients with complete responses (CR) was generally higher across subgroups of interest in the lenvatinib-plus-pembrolizumab arm versus the sunitinib arm (**Figure 3**). As expected, the rates of CRs were higher in patients without baseline bone metastases, and in patients who had a prior nephrectomy. CR rates were similar irrespective of whether or not patients had baseline liver metastases. Among patients with bone metastases, 5 patients in the lenvatinib-plus-pembrolizumab arm had a CR and, of those, 2 patients had received bone-targeting agents.

## 4 Discussion

In this exploratory subgroup analysis of prognostic factors in patients with advanced RCC, efficacy outcomes favored lenvatinib-plus-pembrolizumab versus sunitinib treatment, regardless of presence of baseline lung metastases, bone metastases, liver metastases, prior nephrectomy, or sarcomatoid histology. These findings across PFS, OS, and ORR outcomes are consistent with the efficacy outcomes observed in the intention-to-treat population, in which the efficacy of lenvatinib plus pembrolizumab was superior to that of sunitinib in patients with advanced RCC (2).

Other trials have assessed immune checkpoint inhibitor (ICI)-based combination therapies in patients with advanced RCC (19–22). In the CheckMate 9ER study, with a median follow-up time for OS of 18.1 months, nivolumab plus cabozantinib was superior to sunitinib for PFS (HR 0.51; 95% CI 0.41–0.64), OS (HR 0.60; 98.89% CI 0.40–0.89), and ORR (difference 28.6%) (19). Nivolumab plus cabozantinib was favored over sunitinib for PFS and OS whether or not patients had baseline bone metastases. Nivolumab plus cabozantinib was also favored over sunitinib for PFS irrespective of whether patients had a prior nephrectomy, and was favored over sunitinib for OS among patients who had a prior nephrectomy (19). The CheckMate 214 study, at a median follow-up time of 25.2 months, demonstrated that nivolumab plus ipilimumab was superior to sunitinib for OS (HR 0.63; 99.8% CI 0.44–0.89) and ORR (42% versus 27%, *P* < 0.001) (20). Nivolumab plus ipilimumab was favored over sunitinib for OS, for most subgroups, including patients who did not have bone metastases, patients irrespective of liver metastases, patients with lung metastases, and patients regardless of prior nephrectomy (20). Avelumab plus axitinib was superior to sunitinib for PFS (stratified HR 0.69; 95% CI, 0.56–0.84) and ORR (stratified odds ratio 3.10; 95% CI 2.30–4.15) in the JAVELIN Renal 101 study (21), with a median follow-up for OS of 12.0 months and 11.5 months, respectively. Avelumab plus axitinib was favored over sunitinib for PFS among patients who had a prior nephrectomy, and numerically favored among those patients without a prior nephrectomy, though the patient numbers were low (21). While care should be taken in comparing clinical trial data, and relevant biomarker data for prediction of ICI efficacy are limited (23), these results are all from phase 3 trials in patients with advanced RCC that utilize sunitinib as a comparator. Those factors provide some support for cross-trial comparison, and indicate that ICI-based combination treatments often have superior efficacy over sunitinib; this efficacy extends to patients with certain baseline prognostic features (19–21). The results from assessment of prognostic groups within the CLEAR study indicate that lenvatinib plus pembrolizumab showed superior efficacy over sunitinib in subgroups of interest.

This analysis of data from CLEAR was limited as patient numbers were small for some subgroups, particularly the subgroup with sarcomatoid features (ie, n = 28 patients in the lenvatinib-plus-pembrolizumab arm and 21 patients in the sunitinib arm). Small patient numbers and low numbers of events led to wide CIs, especially in the OS analysis. In addition, not all subgroups were stratification factors, though patient numbers were generally similar between treatment arms. These findings are based on the prespecified subgroup analyses in the statistical analysis plan. Another limitation is that they are exploratory in nature and not statistically powered for individual subgroups (24). Because of the limitation of multiple comparisons, the validity of these findings needs to be confirmed in prospective clinical trials.

Despite limitations inherent to subgroup analyses, these analyses can provide valuable information for treatment customization for patients with RCC. The American Society for Clinical Oncology guidelines suggest ICI-based treatments could be considered for patients with RCC with sarcomatoid features (11), as recent data may indicate that tumors with sarcomatoid features could be especially responsive to ICI-based therapies (25). This recommendation is supported by a molecular analysis of renal cancer tumors, which found that tumors with sarcomatoid features were characterized by low angiogenesis and higher immune presence, which could present mechanistic support for use of ICIs in this subgroup of patients (17). The CheckMate 9ER trial found that among patients with bone metastases, nivolumab plus cabozantinib was more efficacious than sunitinib (19). The data in this analysis demonstrate that lenvatinib plus pembrolizumab was also effective in patients with bone metastases. Among patients with bone metastases, median PFS (HR 0.33, 95% CI 0.21–0.52), median OS (HR 0.50, 95% CI 0.30–0.83), and ORR (odds ratio 6.94, 95% CI 3.51–13.74) were all superior with lenvatinib plus pembrolizumab compared with sunitinib; moreover, of the 85 patients with bone metastases in the lenvatinib-plus-pembrolizumab arm, 5 patients had a best overall response of CR by RECIST v1.1.

The primary results (2) of the phase 3 CLEAR study in patients with RCC supported the combination of lenvatinib plus pembrolizumab as a first-line treatment option for patients with advanced RCC. This further analysis of the prognostic factors of baseline lung, bone, or liver metastases, and of prior nephrectomy and sarcomatoid features, indicates that the superior efficacy of lenvatinib plus pembrolizumab is consistent across multiple important subgroups of patients with advanced RCC.

## Data availability statement

The datasets presented in this article are not readily available because of commercial confidentiality. Requests to access the datasets should be directed to Eisai Oncology Medical Information (esi_oncmedinfo@eisai.com).

## Ethics statement

The trial involving human participants was conducted in accordance with the International Council for Harmonisation Good Clinical Practice Guidelines and the principles of the 2013 Declaration of Helsinki. Institutional review boards or independent ethics committees approved the protocol and appropriate related documents. The patients/participants provided their written informed consent to participate in this study.

## Author contributions

Study concept and design: VG, RP, CH, CO, JM, TKC. Data acquisition: all authors. Data analysis: JEB, RP, CH, JM, CO. Data interpretation: all authors. Review of the manuscript drafts, approval of the final version for submission: all authors.
