# Corrigendum: Phase 3 CLEAR study in patients with advanced renal cell carcinoma: outcomes in subgroups for the lenvatinib-plus-pembrolizumab and sunitinib arms

**DOI:** 10.3389/fonc.2023.1343027

**Published:** 2024-03-01

**Authors:** Viktor Grünwald, Thomas Powles, Masatoshi Eto, Evgeny Kopyltsov, Sun Young Rha, Camillo Porta, Robert Motzer, Thomas E. Hutson, María José Méndez-Vidal, Sung-Hoo Hong, Eric Winquist, Jeffrey C. Goh, Pablo Maroto, Tomas Buchler, Toshio Takagi, Joseph E. Burgents, Rodolfo Perini, Cixin He, Chinyere E. Okpara, Jodi McKenzie, Toni K. Choueiri

**Affiliations:** ^1^Clinic for Medical Oncology and Clinic for Urology, University Hospital Essen, Essen, Germany; ^2^Barts Cancer Institute and the Royal Free Hospital, Queen Mary University of London, London, United Kingdom; ^3^Department of Urology, Kyushu University, Fukuoka, Japan; ^4^State Institution of Healthcare Regional Clinical Oncology Dispensary, Omsk, Russia; ^5^Department of Internal Medicine, Yonsei Cancer Center, Yonsei University Health System, Seoul, Republic of Korea; ^6^Department of Biomedical Sciences and Human Oncology, University of Bari ‘A. Moro’, Bari, Italy; ^7^Department of Medicine, Memorial Sloan Kettering Cancer Center, New York, NY, United States; ^8^Medical Oncology, Texas Oncology, Dallas, TX, United States; ^9^Department of Oncology, Maimonides Institute for Biomedical Research of Córdoba (IMIBIC) Hospital Universitario Reina Sofía, Córdoba, Spain; ^10^Department of Urology, Seoul St. Mary’s Hospital, The Catholic University of Korea, Seoul, Republic of Korea; ^11^Department of Oncology, University of Western Ontario, London, ON, Canada; ^12^ICON Research, South Brisbane & University of Queensland, St Lucia, QLD, Australia; ^13^Department of Medical Oncology, Hospital de la Santa Creu i Sant Pau, Barcelona, Spain; ^14^Department of Oncology, Charles University and Thomayer University Hospital, Prague, Czechia; ^15^Department of Urology, Tokyo Women’s Medical University, Tokyo, Japan; ^16^Global Clinical Development, Merck & Co., Inc., Rahway, NJ, United States; ^17^Clinical Research, Merck & Co., Inc., Rahway, NJ, United States; ^18^Biostatistics, Eisai Inc., Nutley, NJ, United States; ^19^Clinical Research, Eisai Ltd., Hatfield, United Kingdom; ^20^Clinical Research, Eisai Inc., Nutley, NJ, United States; ^21^Department of Medical Oncology, Dana-Farber Cancer Institute, Boston, MA, United States

**Keywords:** renal cell carcinoma, lenvatinib, pembrolizumab, sunitinib, bone metastases, liver metastases, lung metastases, sarcomatoid histology

In the published article, there was an error in the legend for **Figure 3** and Supplementary Table 1 as published. Clarification that objective response rates and complete response rates were calculated based on the number of patients in each listed subgroup was omitted. The corrected legend of both appears below.

**Figure 3:**
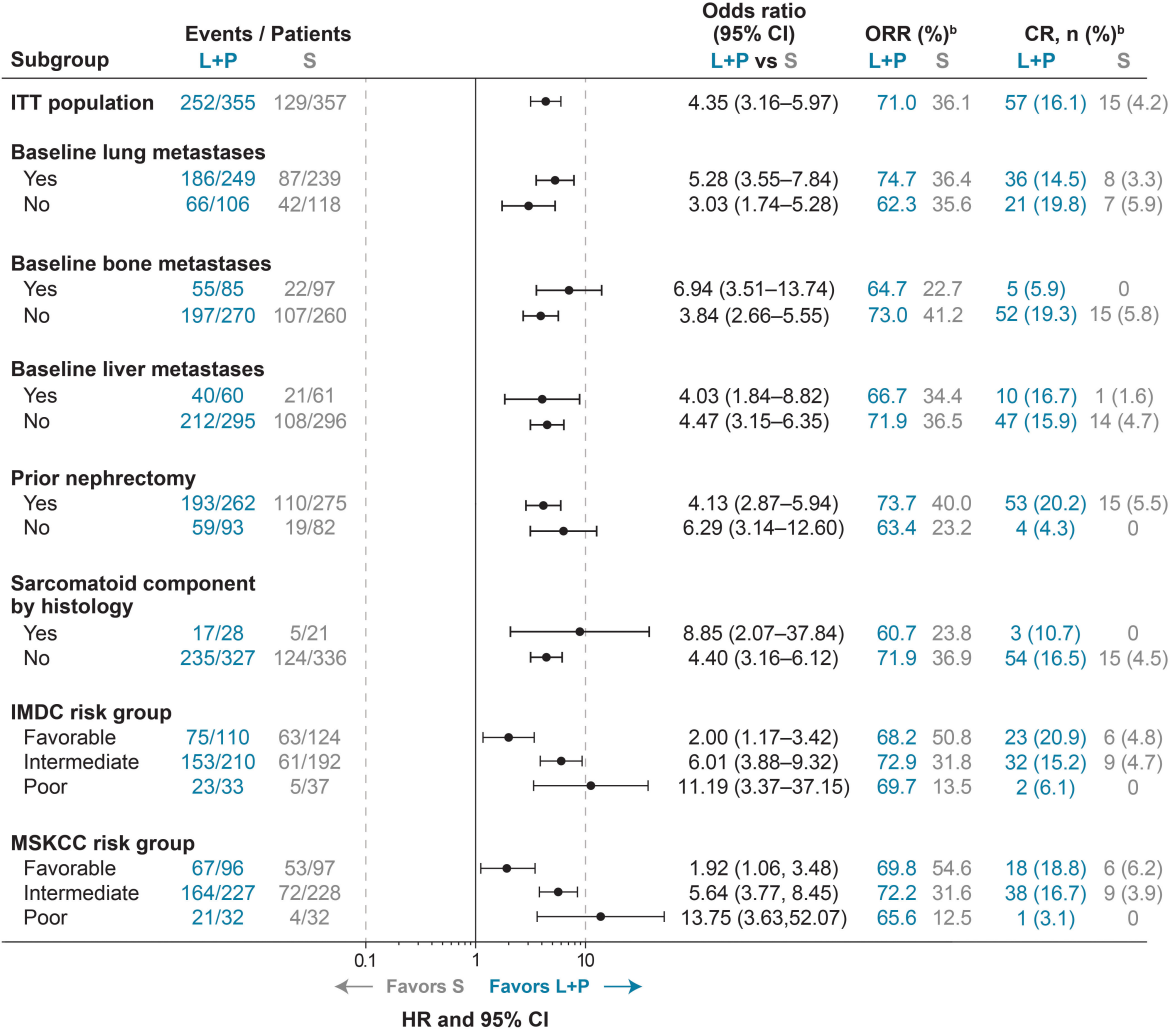
ORR^a^ and Odds Ratios for Lenvatinib + Pembrolizumab Versus Sunitinib Treatment in Subgroups of Interest. ^a^As assessed by IRC per RECIST v1.1. ^b^Percents were calculated based on listed subgroups. CI, confidence interval; CR, complete response; IMDC, International Metastatic Renal Cell Carcinoma Database Consortium; IRC, independent review committee; ITT, intention to treat; L+P, lenvatinib + pembrolizumab; MSKCC, Memorial Sloan Kettering Cancer Center; ORR, objective response rate; RECIST v1.1, Response Evaluation Criteria In Solid Tumors version 1.1; S, sunitinib.

“^a^As assessed by IRC per RECIST v1.1. ^b^Percents were calculated based on listed subgroups.”

In the published article, there was an error in **Figure 3** and Supplementary Table 1 as published. For both, percentages in the “complete response” column were incorrectly calculated as the number of patients with a complete response in each subgroup divided by all patients in the respective treatment arm (n=355 for lenvatinib + pembrolizumab; n=357 for sunitinib) instead of being divided by the number of patients in each applicable subgroup.

The corrected **Figure 3** and its captions (also corrected per the above to “^a^As assessed by IRC per RECIST v1.1. ^b^Percents were calculated based on listed subgroups.”) appear below.

The text did not account for the revisions made to the complete response rate mentioned above.

A correction has been made to Section 3.2.3 (*Objective response*), paragraph 2. This sentence previously stated:

“As expected, the rates of CRs were higher in patients without baseline bone metastases or baseline liver metastases, in patients who had baseline lung metastases, and in patients who had a prior nephrectomy. While the number of patients with sarcomatoid features was small, the rates of CRs in patients without sarcomatoid features was higher than those of patients with sarcomatoid features.”

The corrected sentence appears below:

“As expected, the rates of CRs were higher in patients without baseline bone metastases, and in patients who had a prior nephrectomy. CR rates were similar irrespective of whether or not patients had baseline liver metastases.”

The Supplementary Table 1 has been updated directly in the original article.

The authors apologize for these errors and state that this does not change the scientific conclusions of the article in any way. The original article has been updated.

