# Supplementary material for: Phase 3 CLEAR study in patients with advanced renal cell carcinoma: outcomes in subgroups for the lenvatinib-plus-pembrolizumab and sunitinib arms
Source: Front Oncol. 2023 Aug 16;13:1223282. doi: 10.3389/fonc.2023.1223282 (PMC10471185; doi:10.3389/fonc.2023.1223282)
Supplement: Supplementary file 1 [file Table_1.docx]

**Supplementary Table 1.** ORR^a^ and Odds Ratios for Lenvatinib Plus Pembrolizumab Versus Sunitinib Treatment in Subgroups of Interest

| Parameter | Events/patient | | CR,^a^ n (%)^b^ | | ORR,^a^ (%)^b^ | | Odds ratio (95% CI) |
| --- | --- | --- | --- | --- | --- | --- | --- |
|  | L+P | S | L+P | S | L+P | S |  |
| ITT population | 252/355 | 129/357 | 57 (16.1) | 15 (4.2) | 71.0 | 36.1 | 4.35 (3.16–5.97) |
| Lung metastases | | | | | | | |
| Yes | 186/249 | 87/239 | 36 (14.5) | 8 (3.3) | 74.7 | 36.4 | 5.28 (3.55–7.84) |
| No | 66/106 | 42/118 | 21 (19.8) | 7 (5.9) | 62.3 | 35.6 | 3.03 (1.74 – 5.28) |
| Bone metastases | | | | | | | |
| Yes | 55/85 | 22/97 | 5 (5.9) | 0 | 64.7 | 22.7 | 6.94 (3.51–13.74) |
| No | 197/270 | 107/260 | 52 (19.3) | 15 (5.8) | 73.0 | 41.2 | 3.84 (2.66–5.55) |
| Liver metastases | | | | | | | |
| Yes | 40/60 | 21/61 | 10 (16.7) | 1 (1.6) | 66.7 | 34.4 | 4.03 (1.84–8.82) |
| No | 212/295 | 108/296 | 47 (15.9) | 14 (4.7) | 71.9 | 36.5 | 4.47 (3.15–6.35) |
| Prior nephrectomy | | | | | | | |
| Yes | 193/262 | 110/275 | 53 (20.2) | 15 (5.5) | 73.7 | 40.0 | 4.13 (2.87–5.94) |
| No | 59/93 | 19/82 | 4 (4.3) | 0 | 63.4 | 23.2 | 6.29 (3.14–12.60) |
| Sarcomatoid features | | | | | | | |
| Yes | 17/28 | 5/21 | 3 (10.7) | 0 | 60.7 | 23.8 | 8.85 (2.07–37.84) |
| No | 235/327 | 124/336 | 54 (16.5) | 15 (4.5) | 71.9 | 36.9 | 4.40 (3.16–6.12) |
| IMDC risk group | | | | | | | |
| Favorable | 75/110 | 63/124 | 23 (20.9) | 6 (4.8) | 68.2 | 50.8 | 2.00 (1.17–3.42) |
| Intermediate | 153/210 | 61/192 | 32 (15.2) | 9 (4.7) | 72.9 | 31.8 | 6.01 (3.88–9.32) |
| Poor | 23/33 | 5/37 | 2 (6.1) | 0 | 69.7 | 13.5 | 11.19 (3.37 – 37.15) |
| MSKCC risk group | | | | | | | |
| Favorable | 67/96 | 53/97 | 18 (18.8) | 6 (6.2) | 69.8 | 54.6 | 1.92 (1.06–3.48) |
| Intermediate | 164/227 | 72/228 | 38 (16.7) | 9 (3.9) | 72.2 | 31.6 | 5.64 (3.77–8.45) |
| Poor | 21/32 | 4/32 | 1 (3.1) | 0 | 65.6 | 12.5 | 13.75 (3.63–52.07) |

^a^As assessed by IRC per RECIST v1.1. ^b^Percents were calculated based on listed subgroups.

CI, confidence interval; CR, complete response; IMDC, International mRCC Database Consortium; IRC, independent review committee; ITT, intention to treat; L+P, lenvatinib + pembrolizumab; MSKCC, Memorial Sloan Kettering Cancer Center; ORR, objective response rate; RECIST v1.1, Response Evaluation Criteria In Solid Tumors version 1.1; S, sunitinib.
